# Genomic signatures of seed mass adaptation to global precipitation gradients in sorghum

**DOI:** 10.1038/s41437-019-0249-4

**Published:** 2019-07-17

**Authors:** Jianan Wang, Zhenbin Hu, Hari D. Upadhyaya, Geoffrey P. Morris

**Affiliations:** 1 Department of Agronomy, Kansas State University, Manhattan, KS 66506 USA; 2 Center of Excellence for Advanced Materials Research, King Abdulaziz University, Jeddah, 21589 Saudi Arabia; 3 International Crops Research Institute for the Semi-Arid Tropics, Patancheru, 502 324 India

**Keywords:** Agricultural genetics, Evolutionary biology

## Abstract

Seed mass is a key component of adaptation in plants and a determinant of yield in crops. The climatic drivers and genomic basis of seed mass variation remain poorly understood. In the cereal crop *Sorghum bicolor*, globally-distributed landraces harbor abundant variation in seed mass, which is associated with precipitation in their agroclimatic zones of origin. This study aimed to test the hypothesis that diversifying selection across precipitation gradients, acting on ancestral cereal grain size regulators, underlies seed mass variation in global sorghum germplasm. We tested this hypothesis in a set of 1901 georeferenced and genotyped sorghum landraces, 100-seed mass from common gardens, and bioclimatic precipitation variables. As predicted, 100-seed mass in global germplasm varies significantly among botanical races and is correlated to proxies of the precipitation gradients. With general and mixed linear model genome-wide associations, we identified 29 and 56 of 100 a priori candidate seed size genes with polymorphisms in the top 1% of seed mass association, respectively. Eleven of these genes harbor polymorphisms associated with the precipitation gradient, including orthologs of genes that regulate seed size in other cereals. With FarmCPU, 13 significant SNPs were identified, including one at an a priori candidate gene. Finally, we identified eleven colocalized outlier SNPs associated with seed mass and precipitation that also carry signatures of selection based on *F*_ST_ scans and PCAdapt, which represents a significant enrichment. Our findings suggest that seed mass in sorghum was shaped by diversifying selection on drought stress, and can inform genomics-enabled breeding for climate-resilient cereals.

## Introduction

Local adaptation exists when the average fitness of organisms in their local environment is higher than conspecifics from other environments (Des Marais et al. 2013; Blanquart et al. 2013). Long-term selection acting on phenotypic traits can lead to adaptive divergence, characterized by the divergent morphological characteristics and allele frequencies among local populations (Hoban et al. 2016). Climate is a major factor shaping local adaptation in plants, and the genomic basis of climate adaptation is beginning to be revealed (Eckert et al. 2010; Fournier-Level et al. 2011; Lasky et al. 2012; Kort et al. 2014; Siepielski et al. 2017).

Life-history theory suggests a trade-off between seed mass and seed number, given a constant amount of available resources to produce the seeds (Smith and Fretwell 1974). Increasing seed size provides more reserves for seedling growth, but greater number of seeds provides more propagules and bet-hedging (Olofsson et al. 2009). In semi-arid and arid regions, precipitation-mediated water availability may act as a selective pressure on seed size (Hallett et al. 2011), but findings on seed size adaptation to drought risk have been contradictory (Leishman et al. 2000). In crops, which evolve under both natural and artificial selection, grain size is a main component determining grain yield, subject to similar ecophysiological trade-offs (Sadras 2007).

Sorghum [*Sorghum bicolor* (L.) Moench] is a cereal crop grown in smallholder and commercial agricultural systems in drought-prone regions worldwide (Monk et al. 2014). Sorghum was domesticated in Africa (Wendorf et al. 1992), and diffused widely across diverse agroclimatic zones, including arid, semi-arid, and sub-humid regions, in Africa, Asia, and the Americas (Deu et al. 2006; Morris et al. 2013). Botanists have noted that seed size in common-garden experiments is typically inversely correlated to the abundance of precipitation in the environment of origin, both among and within the botanical races of sorghum (Harlan et al. 1972). Consistent with models of ecophysiological trade-offs, a negative correlation between grain mass and grain number has been observed in sorghum (Yang et al. 2010; Burow et al. 2014; Tao et al. 2018).

The molecular genetic basis of seed size variation has been a major target study due to its potential to facilitate yield improvements (Li et al. 2011; Sosso et al. 2015; Ma et al. 2016). In sorghum, several studies have reported quantitative trait loci (QTL) for seed mass using biparental linkage mapping and genome-wide association study (GWAS) (Paterson et al. 1995; Brown et al. 2006; Feltus et al. 2006; Srinivas et al. 2009; Upadhyaya et al. 2012; Zhang et al. 2015; Han et al. 2015; Boyles et al. 2016; Tao et al. 2018), but the quantitative trait nucleotides have not been identified. By contrast, in the model cereal rice (*Oryza sativa*) molecular networks underlying grain size variation have been characterized in detail (Zuo and Li 2014). Comparative studies suggest ancestral regulatory networks may underlie grain size variation (including mass variation) in cereals and other plants (Zhang et al. 2015).

Ecological and population genomic approaches for detecting signatures of local adaptation have progressed with advances in genotyping (Mita et al. 2013; Tiffin and Ross-Ibarra 2014; Forester et al. 2016). Common garden experiments, which bring germplasm from multiple environments into a common environment, can reveal phenotypic and genomic signatures of local adaptation by isolating heritable genotype effects (Savolainen et al. 2013; de Villemereuil et al. 2016). GWAS of phenotypes or environmental factors can be used to locate genomic regions controlling the putative adaptive traits (Bergelson and Roux 2010; Savolainen et al. 2013). Genome scans for differentiation among populations from different environments can identify adaptive loci without explicitly modeling environmental variation (Beaumont and Balding 2004; Martins et al. 2016).

Genomic signatures of climate adaptation have been reported in many widely-distributed crops including maize, barley, soybean, pearl millet, and sorghum (Vigouroux et al. 2011; Abebe et al., 2015; Lasky et al. 2015; Swarts et al. 2017; Bandillo et al. 2017). Seed size variation in sorghum and other crops has been subject to selection during domestication and diversification (Tao et al. 2017). However, whether sorghum seed mass variation is locally adaptive to global precipitation gradient remains unknown. In sorghum, about 20% of global nucleotide variation can be explained by environmental variables (Lasky et al. 2015), but it is not known how much of this variation affects trait variation, and what trait variation is environmentally adaptive. In this study, we tested the hypothesis that sorghum seed mass variation reflects adaptation to precipitation gradients due to genetic variation in ancestral seed size regulators, and found multiple lines of evidence (phenotypic, geographic, and genomic) consistent with this hypothesis.

## Materials and methods

### Genotypic and environmental data

**Fig. 1:**
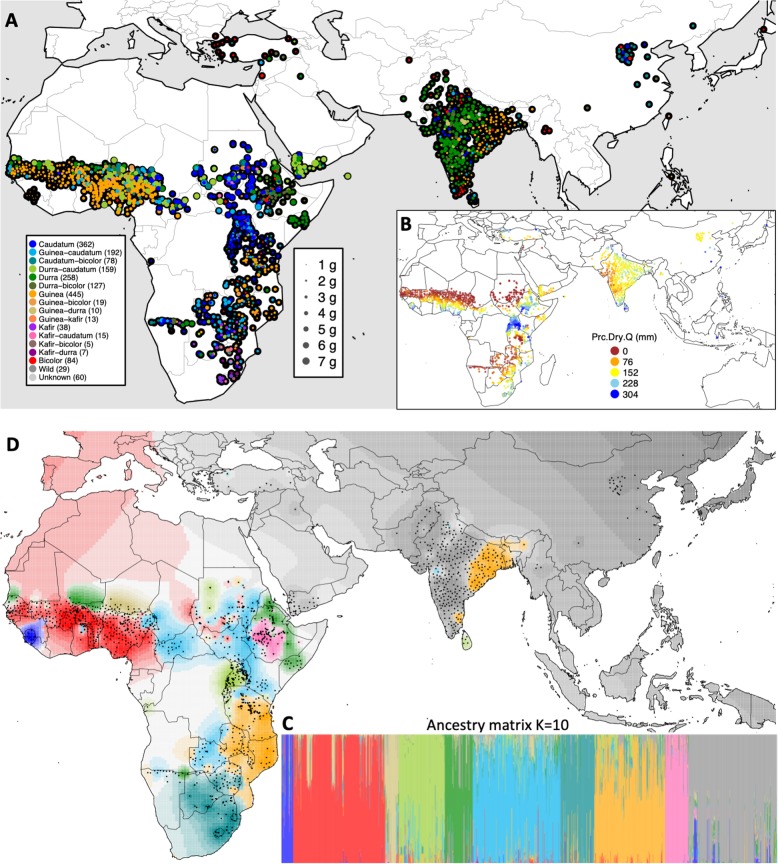
Global diversity panel of sorghum landraces with seed mass variation. **a** Origin of global diverse sorghum landrace accessions and their 100-seed mass in common-garden (BLUPs). Accessions are colored by botanical races (morphological type) with the size of each colored circle proportional to 100-seed mass (with each colored circle plotted on a solid black circle for clarity). **b** The precipitation (precipitation in the driest quarter, in mm) where each sorghum landrace accession originated. **c** The bar plot of sorghum landraces ancestry coefficients based on ADMIXTURE analysis and **d** corresponding spatial population structure map, colored by ancestry coefficients for ten putative ancestral populations (K = 10)

Public genotyping-by-sequencing (GBS) data for 404,627 SNPs and WorldClim bioclimatic variables (Hijmans et al. 2005) for 1901 georeferenced sorghum accessions were obtained from Dryad Digital Repository at 10.5061/dryad.jc3ht (Lasky et al. 2015) (Table S1). The diverse accessions include landraces of all five major botanical races of sorghum and ten intermediate races. In addition, 29 accessions of sorghum wild relatives and 60 accessions with unknown race designations were included. These accessions originated across the majority of the agroclimatic zones in Africa and Asia where sorghum has traditionally been cultivated (Fig. 1a and Table S1).

### Phenotypic data

Seed size was estimated for 1901 sorghum landraces using 100-seed mass obtained from the US National Plant Germplasm System’s Germplasm Resources Information Network (GRIN) or the International Crops Research Institute for the Semi-Arid Tropics (ICRISAT) genebanks (Table S1). Two sets of 100-seed mass data from GRIN (United States) and ICRISAT (India) represent two common-garden experiments. If multiple observations were recorded in GRIN, the mean of all observations for each accession was calculated. To account for the effect of location, best linear unbiased predictors (BLUPs) of 100-seed mass from the two genebanks were estimated for all accessions using a random linear model implemented in R package *lme4* (Bates et al. 2015) as follows:

$$y_{ij} = \mu + G_i + E_j + \varepsilon _{ij}$$

where *y*_*ij*_ is the 100-seed mass of the *i*^th^ accession in the *j*^th^ location, μ is the overall mean, *G*_*i*_ is the random genotypic effect for the *i*^th^ accession, *E*_*j*_ is the random environmental effect of the *j*^th^ location, and ε_*ij*_ is the residual. To test the differences of 100-seed mass among botanical races, one-way analysis of variance (ANOVA) and post hoc Tukey HSD test (TukeyHSD in R) were conducted using 100-seed mass BLUPs.

### Phenotype and precipitation correlations

To characterize the relationship between 100-seed mass BLUPs and each of the five precipitation variables (annual precipitation: Ann.Prc; precipitation in the wettest quarter: Prc.Wet.Q; precipitation in the driest quarter: Prc.Dry.Q; precipitation in the warmest quarter: Prc.Wrm.Q; and precipitation in the coldest quarter: Prc.Cld.Q. The distribution of precipitation variables is skewed, so data were transformed as log_10_(precipitation variable + 1), with the one added to avoid undefined values. Five linear regression analyses were run with 100-seed mass BLUP as dependent variable and each of the five transformed precipitation variables as independent variables using the *lm* function in R package *stats v3.5.0*. A partial correlation analysis using *partial.cor* in R package *RcmdrMisc* was used to estimate the independent effect of each of the five precipitation factors on 100-seed mass variation, while controlling for the remaining four precipitation factors.

### Population genomic analyses

Population structure was characterized with principal component analysis (PCA) using 258,777 SNPs in TASSEL 5.0 (Bradbury et al. 2007), after filtering for SNP with a minor allele frequency >0.02. The genomic ancestry estimates were inferred by a model-based program ADMIXTURE (Alexander et al. 2009), when the pre-defined number range of genetic clusters (K) is from 2 to 17. Since the model in ADMIXTURE does not explicitly take (linkage disequilibrium (LD) into account, a LD-pruned data set of 317,836 SNPs was prepared for ADMIXTURE using *--indep-pairwise 50 5 0.5* parameters in PLINK 1.07 (Purcell et al. 2007). The geographic structure was characterized through spatial mapping of the ancestry coefficients inferred by ADMIXTURE, along with the longitude and latitude coordinates of each accession, using R package *tess3r* (Caye et al. 2016; Martins et al. 2016). LD was calculated using PopLDdecay 3.30 with *-MAF 0.05* for minimum minor allele frequency filtering and *-MaxDist 500* for maximum distance (500 kb) between two SNPs (Zhang et al. 2018). The squared correlation coefficients (*r*^*2*^) were used to estimate LD across the whole population and for each major botanical race. Smoothed LD decay curves were fitted by the *spline* function in R.

### Genome-wide association study of 100-seed mass and precipitation variables

A total of 146,130 biallelic SNPs with minor allele frequency (MAF) >0.05 were used for phenotypic GWAS and environmental GWAS, as a genome scan for local adaptation. A general linear model (GLM; no kinship or structure term), a mixed linear model (MLM) (Yu et al. 2006), and a fixed and random model circulating probability unification (FarmCPU) (Liu et al. 2016) in the R package GAPIT (Lipka et al. 2012) were performed to scan for genome-wide associations between SNPs and BLUPs of sorghum 100-seed mass. For MLM, *model.selection* *=* *TRUE* was selected to determine the optimal number of PCs, which was zero. For FarmCPU, *PCA.total* *=* *5* was used. The individual genetic relatedness matrix (K), which was calculated based on VanRaden method (VanRaden 2008) using the default setting for the *kinship.algorithm* option in GAPIT. For GLM, a threshold at the top 1% smallest *P*-values was set to identify the outlier SNPs for 100-seed mass and precipitation analyses. Given that the nominal *P*-values for GLM are expected to be inflated, we reported the percentiles for the outlier SNPs. For MLM, Bonferroni correction of 3.4 × 10^−7^ (α/number of markers, where α = 0.05) and 1% outlier thresholds were used. Due to the nature of the model, Bonferroni correction of 3.4 × 10^−7^ threshold was used but the top 1% smallest *P*-values threshold was not used for FarmCPU. To examine the degree of model fitness, quantile-quantile (Q–Q) plots of the GWAS models were constructed via plotting quantile distribution of observed −log_10_ (*p*) on the *y*-axis versus the quantile distribution of expected −log_10_(*p*) on *x*-axis.

### Genome-wide scans for local adaptation

Two approaches were applied to screen the genomic signatures of adaptation based on allele frequencies. First, an ancestral allele frequency differentiation test was employed to detect the genome-wide outlier loci that could be under selection using the *snmf* function (Martins et al. 2016) in R package *LEA* (Frichot and François 2015). This method can compute fixation indices (*F*_ST_) and cope with the ambiguous subpopulations simultaneously. Because the assignments of genetic clusters captured most of the genetic variations among different genetic clusters when the number of principal components (K) = 10, the number of genetic clusters was specified as 10, and then we identified outlier loci at this level. SNPs with the top 1% smallest *P*-values were considered outliers. Second, a PCA-based statistic was utilized to detect outlier SNPs that may be involved in local adaptation using R package *pcadapt* (Luu et al. 2017). The PCAdapt method assumes that SNPs excessively related with population structure are candidates for local adaptation, and the PCAdapt method can also deal with admixed individuals without any pre-defined populations. As with SNMF-based *F*_ST_, K was specified as 10.

### A priori candidate genes for seed mass variation

A total of 100 a priori candidate genes of sorghum seed mass on *Sorghum bicolor* were considered in this study, including a published set of 99 orthologs of known cereal seed mass/size genes (Tao et al. 2017) and one sorghum gene Sb04g015420 mapped from a seed size mutant (Jiao et al. 2016). The extent of LD decay (150 kb) was applied to identify the a priori candidate genes of sorghum seed mass, which are nearby the outlier SNPs derived from the genome-wide association studies of 100-seed mass and precipitation, and the selection scans. All genes mentioned in the study were a priori candidates, unless they are noted as post hoc candidates.

### Enrichment analysis

The colocalized outlier SNPs detected through GLM of 100-seed mass, GLM of Prc.Dry.Q, SNMF-based *F*_ST_, or PCAdapt were compared to the set of a priori 100 seed mass candidate genes. First, enrichment analysis for the colocalized top 1% outlier SNPs detected from GLM of 100-seed mass BLUPs and GLM of Prc.Dry.Q was performed. Enrichment of colocalized SNPs was compared to random SNPs derived from the genome-wide 146,130 biallelic SNPs. Second, enrichment analysis for the colocalized top 1% outlier SNPs detected from GLM of 100-seed mass BLUPs, GLM of Prc.Dry.Q and genome-wide scan of selection (SNMF-based *F*_ST_ or PCAdapt) were carried out. Significance of enrichment was tested for the 1% outlier SNPs of GLM of 100-seed mass BLUPs versus GLM of Prc.Dry.Q and the top 1% outlier SNPs detected from the scan of selections (SNMF-based *F*_ST_ or PCAdapt), relative to random SNPs. Both enrichment analyses were calculated using *permTest* function in R package regioneR with the randomization strategy of “resampleRegions” and 1000 permutations (Gel et al. 2016).

## RESULTS

### Characterization of seed mass among different sorghum botanical races

**Fig. 2:**
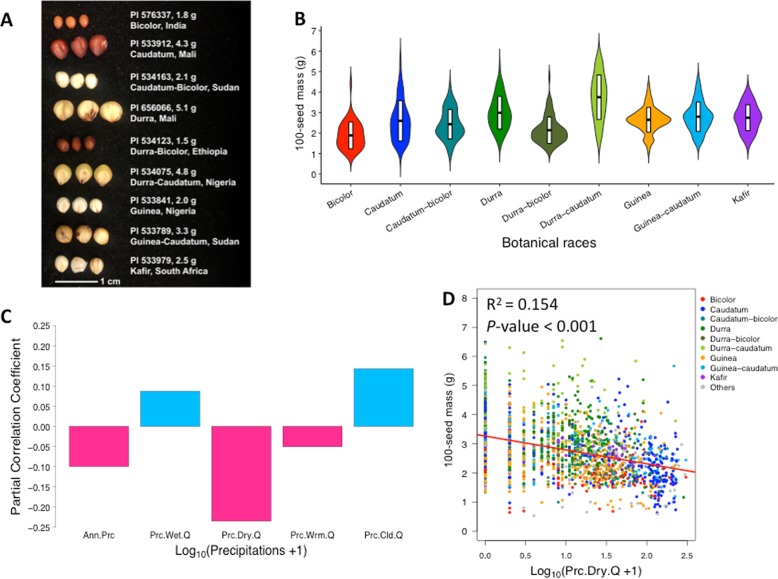
Global variation of common-garden seed mass in sorghum accessions with respect to botanical races and precipitation at the origin. **a** Representative differences of seed mass among sorghum accessions, with accession ID, 100-seed mass, botanical race, and country of origin. **b** Violin plot of 100-seed mass of different sorghum botanical races. Data from nine botanical races in GRIN seed bank and including more than 20 accessions are shown. The means and the standard deviations of 100-seed mass of each botanical races were represented with the black crossbars. **c** Partial correlations between five precipitation variables and 100-seed mass of sorghum accessions. The positive partial correlation coefficients are colored by blue and the negative partial correlation coefficients are colored by red. **d** Correlation of 100-seed mass and log_10_(Prc.Dry.Q + 1) for sorghum accessions. The accessions are colored by botanical race, with botanical races including less than 20 accessions colored in gray

Across the global diversity of georeferenced sorghum accessions, we observed a large variation in 100-seed mass (Fig. 1a; Table 1). The sorghum accessions are classified into five botanical races (bicolor, caudatum, durra, guinea, and kafir) and ten intermediate races based on inflorescence and seed morphology (Harlan et al. 1972). To characterize variations of seed mass among different botanical races and wild relatives of sorghum, we calculated the means of each botanical race and wild species. The georeferenced GRIN accessions used in this study have an average 100-seed mass of 2.7 g, and a range in mass from 0.62 g to 5.8 g (Table 1). Among the nine botanical races with more than 20 accessions, durra-caudatum (3.8 ± 1.08 g) and durra (3.0 ± 0.8 g) sorghums have the heaviest 100-seed mass, while bicolor (1.9 ± 0.65 g) and durra-bicolor (2.1 ± 0.7 g) sorghums have the lightest 100-seed mass (Table 1; Fig. 2a, b). Notably, durra and durra-caudatum sorghums predominate in the most arid portion of the traditional sorghum range (the Sahel, the Arabian peninsula, and the western plateau of India), while durra-bicolor sorghum predominates in the humid highlands of Ethiopia (Fig. 1a, b). Bicolor sorghums, which are hypothesized to be an ancestral form (Harlan et al. 1972), have no notable geographic distribution. There is a significant difference of 100-seed mass among botanical races (F[16, 1884], *P* < 10^−15^). Post hoc comparisons based on the Tukey HSD test indicated that 41 of 136 pairwise comparisons among botanical race are significant when adjusted *P* < 0.01 (Table S2).

**Table 1:** Summary of variation for 100-seed mass in global sorghum germplasm

Botanical race	Number of accessions	Seed mass (g)^a^		
		GRIN	ICRISAT	BLUP
Bicolor	84	1.9 ± 0.65 [0.9, 4.4]	1.9 ± 0.60 [0.64, 3.8]	−0.73 ± 0.47 [−1.7, 0.54]
Caudatum	362	2.6 ± 0.99 [0.7, 5.8]	2.7 ± 1.0 [0.63, 6.5]	−0.07 ± 0.84 [−1.7, 2.9]
Caudatum-bicolor	78	2.4 ± 0.72 [1.0, 4.2]	2.8 ± 1.0 [0.99, 6.0]	−0.12 ± 0.67 [−1.5, 1.7]
Durra	258	3.0 ± 0.80 [1.5, 5.2]	3.2 ± 0.87 [1.2, 6.6]	0.31 ± 0.67 [−1.3, 3.0]
Durra-bicolor	127	2.1 ± 0.65 [1.2, 4.7]	2.5 ± 0.71 [1.4, 5.4]	−0.36 ± 0.57 [−1.2, 2.1]
Durra-caudatum	159	3.8 ± 1.1 [1.7, 5.7]	3.7 ± 1.2 [1.5, 6.5]	0.79 ± 0.95 [−1.0, 2.9]
Guinea	445	2.7 ± 0.60 [0.94, 4.4]	2.6 ± 0.76 [0.8, 5.0]	−0.11 ± 0.60 [−1.6, 1.7]
Guinea-caudatum	192	2.8 ± 0.72 [1.4, 4.8]	2.9 ± 0.74 [1.2, 5.7]	0.07 ± 0.59 [−1.2, 1.6]
Kafir	38	2.8 ± 0.63 [1.7, 3.9]	2.8 ± 0.43 [1.8, 4.4]	−0.01 ± 0.37 [−0.9, 1.0]
Wild	29	1.8 ± 0.71 [0.6, 3.3]	1.3 ± 0.56 [0.53, 2.5]	−1.1 ± 0.48 [−1.8, 0.11]
Overall	1901	2.7 ± 0.89 [0.6, 5.8]	2.8 ± 0.97 [0.53, 6.6]	0.0 ± 0.77 [−1.8, 3.0]

^a^Given are the mean ± standard deviation, and [minimum, maximum] across accessions for each botanical race

### Relationship between seed mass and precipitation variables

To characterize the relationship between seed mass and precipitation, we correlated 100-seed mass and each of five transformed bioclimatic precipitation variables (n_accession_ = 1897). The correlation of Prc.Dry.Q accounts for 15.4% of the variance of 100-seed mass (*R*^2^ = 0.15, *P* < 0.001), more than the correlation of the other four precipitation variables: Prc.Wrm.Q (*R*^2^ = 0.082, *P* < 0.001), Ann.Prc (*R*^2^ = 0.051, *P* < 0.001), Prc.Cld.Q (*R*^2^ = 0.047, *P* < 0.001), Prc.Wet.Q (*R*^2^ = 0.008, *P* < 0.001) (Fig. 2d; Fig. S1). To further explore the independent effect of each of the five precipitation variables on the 100-seed mass, we conducted a partial correlation analysis. The precipitation variables Prc.Wet.Q (*r* *=* 0.087, adj. *P* = 0.0004) and Prc.Cld.Q (*r* *=* 0.14, adj. *P* < 0.0001) had individually positive partial correlation to 100-seed mass BLUPs. By contrast, Ann.Prc (*r* *=* −0.10, adj. *P* < 0.0001), Prc.Dry.Q (*r* *=* −0.23, adj. *P* < 0.0001), and Prc.Wrm.Q (*r* *=* −0.05, adj. *P* = 0.03) had individually negative partial correlation to 100-seed mass BLUPs. Among the five partial correlation tests, the strongest partial correlation was observed between 100-seed mass BLUPs and the Prc.Dry.Q (*r* *=* −0.23, adj. *P* < 0.0001) (Fig. 2c), so Prc.Dry.Q was targeted in further analyses.

### Genome-wide SNP variation in global georeferenced germplasm

We performed two approaches to characterize the population structure and spatial genetic differentiation of the global sorghum data set. PCA and ancestry analyses showed similar complex hierarchical population structure reflecting geographic origin and botanical race (Fig. 1c, d; Fig. S2A). LD dropped to half its maximum value at ~25 kb and to background level (*r*^2^ < 0.1) at ~150 kb (Fig. S2B). LD substantially varied among the five major botanical races and sorghum wild relatives, and the LD of wild sorghum decayed much faster than the domesticated botanical races. Among domesticated sorghum, bicolor sorghum had the fasted LD decay followed by caudatum, durra, and guinea sorghum (Fig. S2B).

### Genome-wide association studies of seed mass

**Fig. 3:**
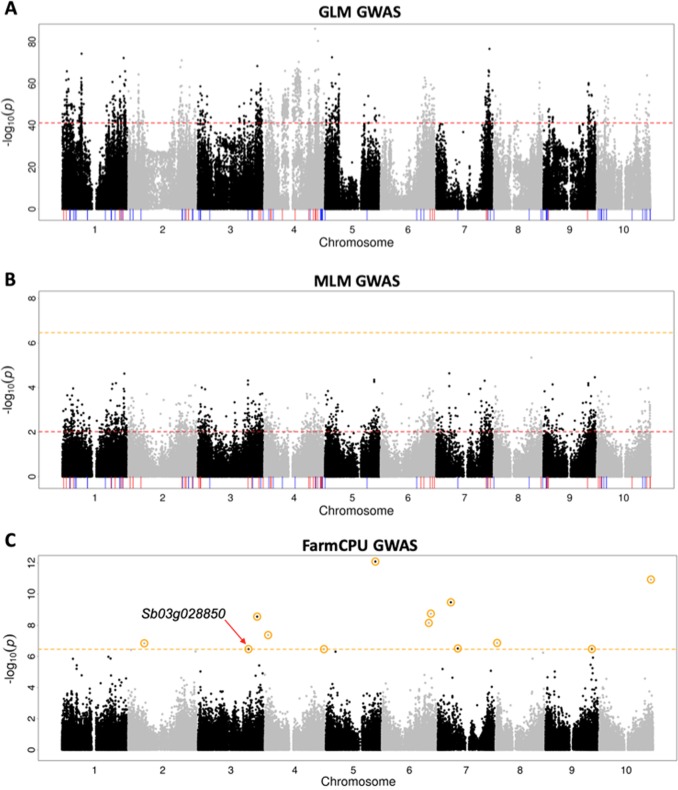
Genome-wide association studies of seed mass. Negative log10 *P*-values are plotted against genome position for **a** GLM, **b** MLM, and **c** FarmCPU GWAS of 100-seed mass BLUPs. Red horizontal dashed line indicates the genome-wide thresholds of the top 1% outlier SNPs with for GLM (−log10(*p*) = 41.4) and MLM (−log10(*p*) = 2.0). Orange horizontal dashed line indicates the genome-wide thresholds of Bonferroni correction (*P* < 0.05) for MLM and FarmCPU (−log10(*p*) = 6.5). Red vertical lines indicate a priori candidate genes, which are within 150 kb from a top 1% outlier SNPs of seed mass for GLM (*n* = 29) or MLM (*n* = 56). Blue lines indicate all other a priori candidate genes. Orange circles indicate 13 outlier SNPs of seed mass and the a priori candidate gene Sb03g028850 (*ZmSh2*) colocalized with the outlier SNP S3_57075248 labeled for FarmCPU

To identify genomic regions associated with seed mass in sorghum, we scanned for associations between SNPs and BLUPs of 100-seed mass using GLM, MLM, and FarmCPU. The Q–Q plots were shown in Fig. S3 A-C. The 1% outlier SNPs for 100-seed mass associations (*n* = 1461) were distributed on all ten chromosomes. Among these SNPs, 96 outlier SNPs were colocalized with 29 a priori candidate genes of seed mass (within 150 kb) on chromosomes 1, 2, 3, 4, 6, 7, and 9 (Fig. 3a; Table S3). The most significant SNP of the 96 outlier SNPs was on chromosome 4 (S4_57856768, MAF = 0.50, 99.9th percentile), ~9 kb from the ortholog of *OsPGL2* (Sb04g027930) (Table S3). Additionally, one of the 29 most significant SNPs (S4_35211218, MAF = 0.14, 98.8th percentile) was in the sorghum mutant seed size gene Sb04g015420 (Table S3; Fig. 6a).

No significant SNPs were identified by MLM with a threshold −log10(*p*) = 6.5 after Bonferroni correction (Fig. 3b). With MLM and top 1% associated SNPs as threshold, we identified associated genomic regions on all ten chromosomes. Among the outlier SNPs, 126 SNPs on the nine chromosomes were colocalized within 150 kb flanking regions of 56 a priori candidate genes of seed mass (Fig. 3b; Table S4). The most significant of the 126 outlier SNPs was on chromosome 3 (S3_57075248, MAF = 0.38, *P* < 10^−4^), 58 kb from the putative ortholog of *ZmSh2* (Sb03g028850) (Table S4). Similarly, one of the 56 most significant SNPs was in a priori gene Sb04g022620, which is the ortholog of *OsSDG725* (Table S4).

Thirteen significant SNPs were identified by FarmCPU with a threshold −log10(*p*) = 6.5 after Bonferroni correction (Fig. 3c; Table S5). The thirteen SNPs were distributed on nine chromosomes, except Chromosome 1. These SNPs were colocalized within 150 kb flanking regions of 293 sorghum genes (Table S6). Among the thirteen SNPs, the twelfth most significant SNP, S3_57075248 (MAF = 0.38, *P* < 10^−6^), is 58 kb from the putative ortholog of *ZmSh2* (Sb03g028850). Sb03g028850 is the only a priori candidate gene of sorghum seed mass included in the detected 293 sorghum genes via FarmCPU.

### Genome-wide association studies of precipitations

**Fig. 4:**
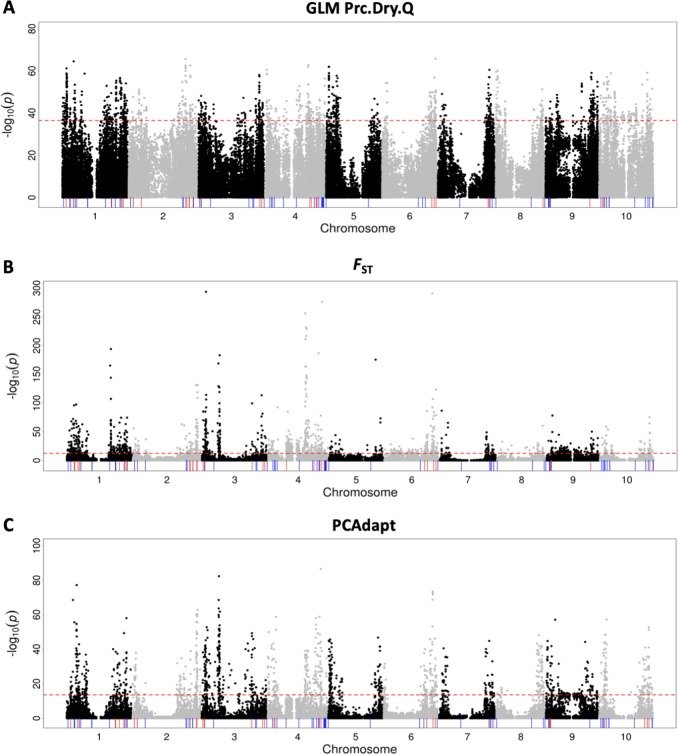
Genome-wide scans for adaptation. Negative log10-transformed *P*-values from the genome-wide SNP scans are plotted against the genome position for **a** GLM association with precipitation in the driest quarter (Prc.Dry.Q), **b** SNMF-based *F*_ST_, and **c** PCAdapt using K = 10. Red horizontal dashed line indicates the genome-wide threshold of the top 1% outlier SNPs for association with Prc.Dry.Q (−log10(*p*) = 36.5), SNMF-based *F*_ST_ (−log10(*p*) = 12.5) and PCAdapt (−log10(*p*) = 13.5). Red vertical lines represent a priori candidate genes of seed mass above the given thresholds for association with Prc.Dry.Q (*n* = 36), SNMF-based *F*_ST_ (*n* = 35), and PCAdapt (*n* = 37). Blue vertical segments indicate the undetected 64, 65, and 63 a priori candidate genes of seed mass below the thresholds for association with Prc.Dry.Q, SNMF-based *F*_ST_, and PCAdapt, respectively

To identify candidate genes of seed mass that are associated with precipitation gradients, we explicitly tested the genome-wide associations between SNPs and precipitation gradients. Because precipitation in the driest quarter (Prc.Dry.Q) exhibited the strongest correlation to the 100-seed mass BLUPs based on both the linear regression analysis and partial correlation analysis, the Prc.Dry.Q was used as the proxy for the precipitation gradient in further analysis. The 1% outlier SNPs for Prc.Dry.Q associations (*n* = 1461) were identified on ten chromosomes. Of these SNPs, 112 were colocalized with the 36 a priori candidate genes on nine chromosomes, excluding chromosome 5 (Fig. 4a; Table S7). The most highly associated SNP that was colocalized with an a priori candidate gene was S2_64282627 (MAF = 0.37, 99.9th percentile). This SNP is 146 kb from candidate seed mass gene Sb02g029300, the ortholog of *OsGW8* (Table S7). No outlier SNPs fell inside an a priori candidate gene. The Q–Q plot was shown in Fig. S3D.

### Genome-wide scans for local adaptation

Genomic regions displaying signatures of selection among populations could suggest adaptation to diverse environmental gradients. We conducted SNMF-based *F*_ST_ and PCAdapt analyses to detect outlier SNPs involving allele frequency differentiation. The top 1% outlier SNPs identified from both methods were distributed on all ten chromosomes. From SNMF-based *F*_ST_, 133 outlier SNPs identified on chromosomes 1, 2, 3, 4, 6, 9, and 10 were colocalized with the 150 kb flanking regions of 35 a priori candidate seed mass genes (Fig. 4b; Table S8). The most significant SNP among the 133 outliers was S6_58925412 (MAF = 0.082, *P* < 10^−23^), which is ~20 kb from the seed mass candidate gene Sb06g030550, an ortholog of *OsFLO2* (Table S8). Additionally, two outlier SNPs fell inside two a priori candidate genes of seed mass on chromosome 3. First, S3_57017386 (MAF = 0.09, *P* < 10^−99^) was located inside Sb03g028850, the ortholog of *ZmSh2* (Table S8). Second, S3_71459932 (MAF = 0.12, *P* < 10^−42^) was in Sb03g044160, the ortholog of *OsGW8* (Table S8).

As a complement to the SNMF-based *F*_ST_ analysis, PCAdapt identified 120 outlier SNPs on eight chromosomes, which were colocalized within 150 kb of 37 a priori candidate genes of seed mass (Fig. 4c; Table S9). The most significant SNP of the 120 outlier SNPs was S2_72877577 (MAF = 0.15, *P* < 10^−60^), ~5 kb away from seed mass candidate gene Sb02g038670, an ortholog of *OsBG2* (Table S9). In accordance with the SNMF-based *F*_ST_ results, S3_71459932 (MAF = 0.12, *P* < 10^−15^) was also detected in PCAdapt, located inside the ortholog of *OsGW8* (Sb03g044160; Table S9).

### Colocalized SNPs and enrichment analysis

**Fig. 5:**
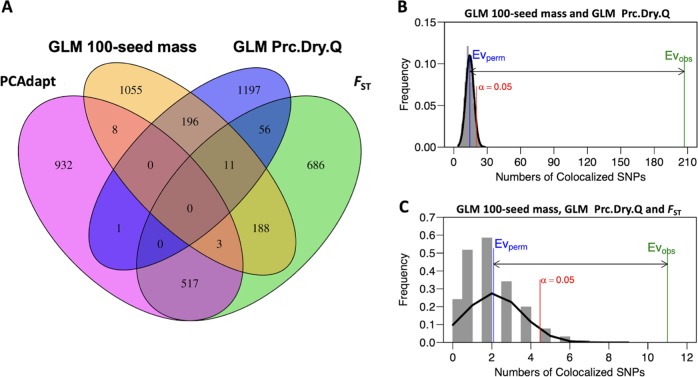
Colocalized outlier SNPs among phenotypic association, environmental association, and selection scans. **a** Venn diagrams of outlier SNPs identified jointly by GLM of 100-seed mass, GLM of precipitation in the driest quarter (Prc.Dry.Q), SNMF-based *F*_ST_, and PCAdapt. Enrichment analysis for **b** seed mass association and precipitation associations, or **c** seed mass associations, precipitation associations, and *F*_ST_. In panel **b**, **c**, the blue line (Ev_perm_) represents the mean of colocalized SNPs from the null distribution with 1000 permutations, the red line represents α = 0.05 for the null distribution, and the green line (Ev_obs_) represents the number of the observed colocalized SNPs

Enrichment of a priori seed mass genes with GWAS and genome scan outliers may provide additional evidence of seed mass adaptation. A total of 207 colocalized outlier SNPs diffusing on the ten chromosomes were identified based on the top 1% outlier SNPs detected by GLM of 100-seed mass BLUPs and GLM of precipitation (Fig. 5a; Table S10). There was a significant enrichment for the colocalized 207 outlier SNPs (*P* < 0.001; Fig. 5b). A total of 11 a priori sorghum seed mass candidate genes on chromosome 1, 2, 3, 4, 6, 7, and 9, which fell into 150 kb flanking the colocalized 207 outlier SNPs, were identified (Table 2). A different set of 11 colocalized outlier SNPs at four loci (chromosomes 1, 4, 7, and 8) were identified based on the top 1% outlier SNPs detected by GLM of 100-seed mass, GLM of precipitation and SNMF-based *F*_ST_ method (Table S11). Seven sorghum genes (not a priori candidates) nearest (<2 kb) to the eleven colocalized outlier SNPs were identified (Table S11). However, no colocalized SNPs were identified based on the top 1% outlier SNPs detected by GLM of 100-seed mass, GLM of precipitation, and PCAdapt (Fig. 5a). There was a significant enrichment for the colocalized eleven outlier SNPs (*P* < 0.001; Fig. 5c). However, no a priori sorghum 100-seed mass candidate genes were identified, because none of them fell into 150 kb flanking the eleven outlier SNPs.

**Table 2:** Eleven a priori sorghum seed mass candidate genes that colocalized with outlier SNPs for both GLM of 100-seed mass and GLM of Prc.Dry.Q

SNP ID^a^	Gene^b^	Gene Start^b^	Gene End^b^	Gene^c^	Ortholog^d^
S1_4315232	Sb01g005170	4275513	4279485	Sobic.001G056700	ZmO2
S1_65251930	Sb01g041900	65135319	65141117	Sobic.001G445900	OsCYP90B1
S2_65532747	Sb02g030640	65662195	65664496	Sobic.002G272700	AtEOD3
S2_68333169	Sb02g033830	68370266	68371629	Sobic.002G308400	MYB
S2_68333169	Sb02g033940	68469057	68478741	Sobic.002G309600	AtUPF1
S3_69399186	Sb03g042060	69485282	69485858	Sobic.003G380900	OsSERF1
S4_50682434	Sb04g021540	50589773	50601467	Sobic.004G163700	ZmSbeIIb
S4_58788981	Sb04g028690	58829498	58833664	Sobic.004G247000	ZmGln-4
S6_56374341	Sb06g027465	56363893	56367340	Sobic.006G203400	OsGS2
S7_57962621	Sb07g023040	57901670	57902598	Sobic.007G156800	OsSGL1
S9_5395395	Sb09g004510	5453946	5459014	Sobic.009G053600	OsGS5

^a^SNP ID includes the chromosome number (before the underscore) and position (after the underscore) in v1.4 coordinates

^b^Gene name and position in v1.4 sorghum genome assembly

^c^Gene name in v3.0 sorghum genome assembly

^d^Details on orthologs in ref. (Tao et al. 2017)

## Discussion

### Evidence for clinal adaptation of seed mass to precipitation gradients

Sorghum has wide variation in seed mass, over 7-fold among accessions of domesticated sorghum (Fig. 1a; Fig. 2a, b; Table 1). This observation runs counter to the literature on wild plants, which suggests that within-species variation in seed mass is small (<4-fold) relative to among-species and within-plant variation (Silvertown 1989; Leishman et al. 2000). While the genebank 100-seed mass data do not capture within-plant or among-plant variation for a given genotype, we do not expect these to be major contributors to the observed variance, since genebank seed is produced under near-optimal conditions where stress and microenvironment variation is minimized (Tao et al. 2018).

Ecological studies of wild plants suggest that plants adapted to drought-prone environments produce larger seeds (Baker 1972; Hallett et al. 2011), but whether this is a general phenomenon remains contentious (Leishman et al. 2000). In the present study, the significant differences of seed mass among sorghum botanical races from precipitation zones (Fig. 2a-d; Fig. S1 & S2) support the hypothesis that the precipitation gradient has shaped genetic variation of seed mass within and among botanical races of sorghum. For instance, durra sorghum, adapted to arid climates of Sahelian West Africa and western India, produce larger seeds than guinea sorghums, adapted to subtropical high rainfall climate zone in the Guinean West Africa and eastern India (Fig. 1a, b).

Based on our findings and previous studies, we hypothesize that large seeds are favored in arid agroclimatic zones due to a “seedling size effect”, where additional reserves in larger seeds help seedlings survive drought stress (Leishman et al. 2000). Given that wild sorghums have small seeds (Table 1), we hypothesize, parsimoniously, that small-seeded domesticated sorghum in humid agroclimatic zones reflect the ancestral state, rather than diversifying selection for reduced seed size in domesticated sorghum. We note that other environmental variables are correlated with precipitation across the global distribution of sorghum (Lasky et al. 2015). Therefore, further population and quantitative trait analyses will be required to confirm these hypotheses, and disentangle adaptation to precipitation from adaptation to temperature, soil, or other factors.

### Genomic signatures of seed mass regulation and precipitation adaptation

**Fig. 6:**
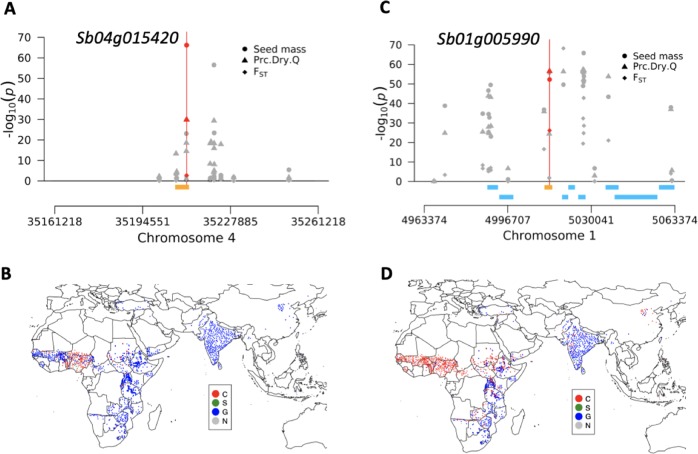
Seed mass and precipitation associations of alleles in two candidate genes. **a** A regional Manhattan plot around confirmed sorghum seed mass gene Sb04g015420 (orange bar). The GLM of 100-seed mass identified the outlier SNP S4_35211218 (red circle) inside the gene. However, the outlier SNP S4_35211218 was not identified as a significant SNP by GLM of precipitation (red triangle point) and SNMF-based *F*_ST_ (red diamond). **c** A regional Manhattan plot around post hoc candidate gene Sb01g005990 (orange bar), which putatively encodes a glutathione S-transferase. GLM of 100-seed mass, GLM of precipitation and SNMF-based *F*_*ST*_ all identified the outlier SNP S1_5013374 (represented by red circle, triangle point, and diamond, respectively) in the gene. Global allele distribution for **b** S4_35211218 and **d** S1_5013374, with color code indicating the genotype for each given accession

To our knowledge, no natural variants controlling seed mass or size in sorghum have been identified (Tao et al. 2017, 2018). A top association from GLM GWAS of seed mass (S4_35211218) fell within a confirmed sorghum seed size and mass gene Sb04g015420 (Jiao et al. 2016), suggesting that this gene identified via mutant analysis also underlies natural variation in seed mass (Fig. 6a, b). Both the maize ortholog *ZmSWEET4c* and rice ortholog *OsSWEET4* of Sb04g015420 regulate the grain filling via SWEET-mediated hexose transport (Sosso et al. 2015). Among the candidate genes identified by the seed mass GWAS, several (15/29 from GLM, 16/56, from MLM) have further evidence from colocalization with linkage mapping QTL intervals (Table S12). For example, Sb09g004510, an ortholog of *GS5*, colocalized with the interval of the sorghum grain weight QTL SDWT.LG9-2 (Brown et al. 2006). In rice and wheat, *GS5* regulates seed size by promoting cell division (Li et al. 2011; Ma et al. 2016), so Sb09g004510 may play a similar role in sorghum. FarmCPU GWAS identified a promising a priori candidate gene of seed mass, Sb03g028850, which is a putative ortholog of *ZmSh2* involving starch biosynthesis. Sb03g028850 was also identified by the MLM GWAS and with top 1% most significant association as the threshold.

Several lines of evidence in the present study supported the hypothesis that genetic variation around sorghum loci regulating seed mass was shaped by adaptation to precipitation gradients. Eleven a priori seed mass candidate genes were identified simultaneously from genomic signals of seed mass association and Prc.Dry.Q association (Fig. 5a, b; Table S11; Table 1), consistent with precipitation gradients shaping seed mass variation. Sb02g033830 (MYB67) is an ortholog of a MYB transcription factor in rice that was identified from a grain yield GWAS (Huang et al. 2012). Sb01g041900 is an ortholog of *OsCYP90B1* and *OsCYP90B2*, brassinosteroid biosynthesis genes that regulate grain filling in rice (Wu et al. 2008). The seven nearest sorghum genes (not a priori candidates) to the eleven colocalized outlier SNPs may be considered post hoc candidate genes for seed mass adaptation (Table S12). Among the seven genes, Sb01g005990 (Fig. 6c, d) and Sb01g006000 putatively encode glutathione S-transferase (GST). GSTs have been implicated in drought tolerance in *Arabidopsis* (Chen et al. 2012), but to our knowledge no studies have suggested a role for GSTs in seed mass.

Identification of the overlapping genomic regions between positional and association genetic studies can further support the results of each method (Zhang et al. 2015). The GLM and MLM of seed mass identified 29 and 56 a priori candidate genes of seed mass (Table S3 & S4), respectively, suggesting the diverse mechanisms could regulate sorghum seed mass. For example, *OsPGL2*, the ortholog of Sb04g027930, is a bHLH transcription factor that positively regulates the rice grain length (Heang and Sassa 2012). Similarly, *OsSDG725*, the ortholog of Sb04g022620, is H3K36 methyltransferase that regulates brassinosteroid-dependent grain development in rice (Sui et al. 2012). Further functional investigations of these candidate genes will be required to confirm effects on seed mass and test for any role in drought adaptation.

### Prospects for dissection and improvement of climate-adaptive seed mass variation

Identifying the most effective genomic approaches to dissect adaptive trait variation is an ongoing challenge (Mita et al. 2013; Tiffin and Ross-Ibarra 2014; Lipka et al. 2015; Forester et al. 2016; Bouchet et al. 2017). In this study, few outlier SNPs were colocalized among all methods (Fig. 5a). Similar findings were reported in a study of local adaptation in the wild progenitor of soybean *Glycine soja* (Bandillo et al. 2017). Different assumptions of each method may be one reason for the small number of colocalized outlier SNPs (Akey 2009).

Population structure observed in our study reflected highly structured global sorghum diversity, which can confound association approaches (Fig. 1c, d; Fig. S2A) (Morris et al. 2013). The geographic patterns and GWAS findings indicate seed mass is a trait that is strongly confounded with population structure (Fig. 3a). Although the MLM controlled inflation efficiently (Fig. 3b), no significant association outliers were identified by MLM at the Bonferroni threshold, suggesting false negatives due to a collinear polygenic and oligogenic components of variance (Myles et al. 2009; Lipka et al. 2015). As shown in the Q–Q plots (Fig. S3 A-C), FarmCPU model is best fitted our 100-seed mass data. The FarmCPU in our 100-seed mass GWAS study can relieve the confounding effect efficiently, such as populations structure, and strengthen the statistical power. The performance of FarmCPU in our study supported its developer’s statement on improved power (Liu et al. 2016). To break up population structure and gain mapping power, multi-parental mapping with multi-advanced generation intercross (MAGIC) or nested association mapping (NAM) populations should be an effective approach (Bouchet et al. 2017; Ongom and Ejeta 2018).

The phenotypic, geographic, and genomic signatures we characterized here provide new evidence that seed mass variation can be adaptive to precipitation gradients (Leishman et al. 2000). In addition to basic insights on ecology and evolution, these findings may help crop improvement programs to identify germplasm harboring adaptive alleles for seed mass and drought tolerance. Genomics knowledge on adaptive and agronomic traits can ultimately be leveraged into genomics-enabled breeding of climate-resilience in sorghum and other crops (Zuo and Li 2014; Tao et al. 2017).

## Supplementary information

Supplemental Material

TableS1

TableS2

TableS3

TableS4

TableS5

TableS6

TableS7

TableS8

TableS9

TableS10

TableS11

TableS12

## Data Availability

All data are publicly available. SNP genotype, geographic, and precipitation data are available from Dryad Digital Repository at 10.5061/dryad.jc3ht and provided in Table S1. The 100-seed mass data from GRIN, ICRISAT, and BLUPs data can be accessed in Table S1.
