# Supplementary material for: Genomic signatures of seed mass adaptation to global precipitation gradients in sorghum
Source: Heredity (Edinb). 2019 Jul 17;124(1):108–21. doi: 10.1038/s41437-019-0249-4 (PMC6906510; doi:10.1038/s41437-019-0249-4)
Supplement: Supplementary file 1 — Supplemental Material [file 41437_2019_249_MOESM1_ESM.docx]

**Supplemental Material**

**Genomic signatures of seed mass adaptation to global precipitation gradients in sorghum**

Jianan Wang^1^, Zhenbin Hu^1^, Hari D. Upadhyaya^1,2^, *Geoffrey P. Morris^1^

^1^ Department of Agronomy, Kansas State University, Manhattan, KS 66506, USA; ^2^ Center of Excellence for Advanced Materials Research, King Abdulaziz University Jeddah 21589, Saudi Arabia.

Author for correspondence:

*Geoffrey P. Morris*

*Tel: 1-785-532-3397*

*Email: gpmorris@ksu.edu*

Running Head: Seed mass adaptation to precipitation in sorghum

**This file includes:** Figures S1-S3; and Tables S1-S12.

**
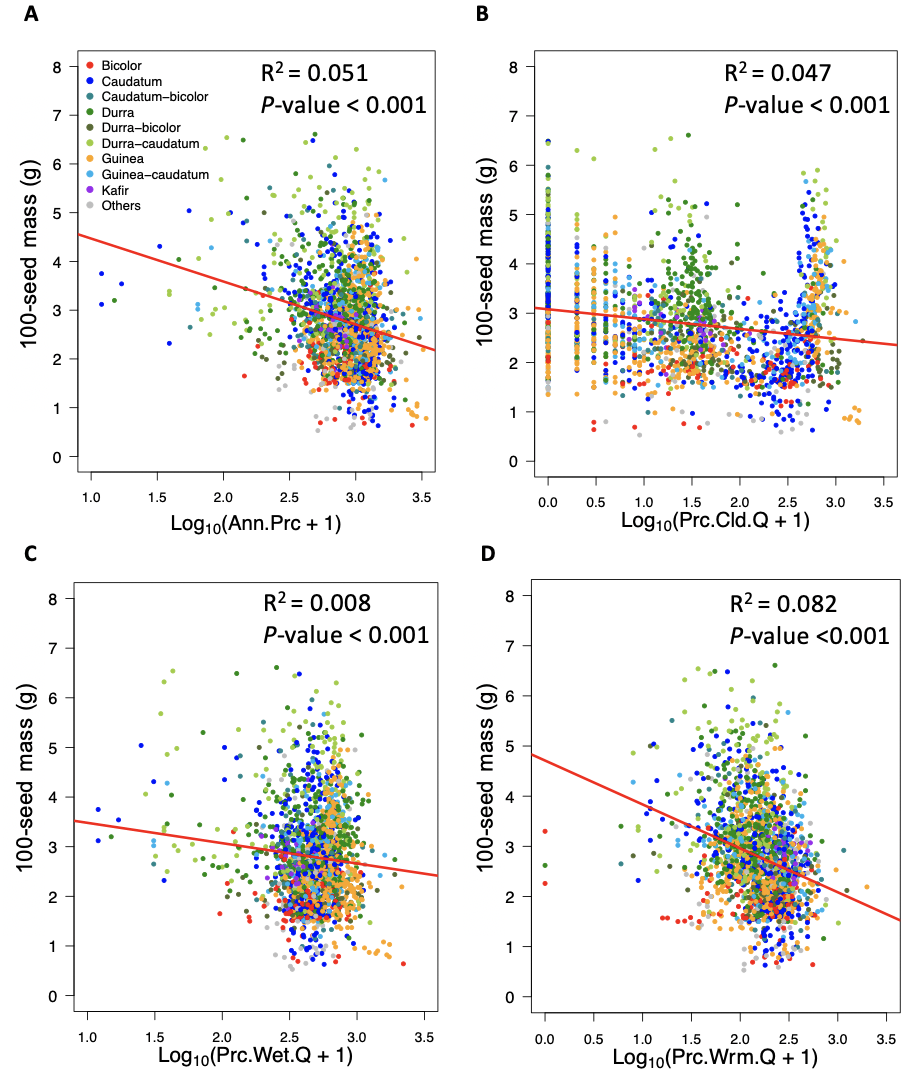
**

**Figure S1 Correlations between four different precipitation variables Log_10_(Precipitations +1) and 100-seed mass of sorghum landraces.** (A) Annual precipitation (Ann.Prc). (B) Precipitation in the coldest quarter (Prc.Cld.Q). (C) Precipitation in the wettest quarter (Prc.Wet.Q). (D) Precipitation in the warmest quarter (Prc.Wrm.Q). The four panels shared the same color scheme. The landrace are colored by botanical races. All botanical races including less than 20 accessions are grey.


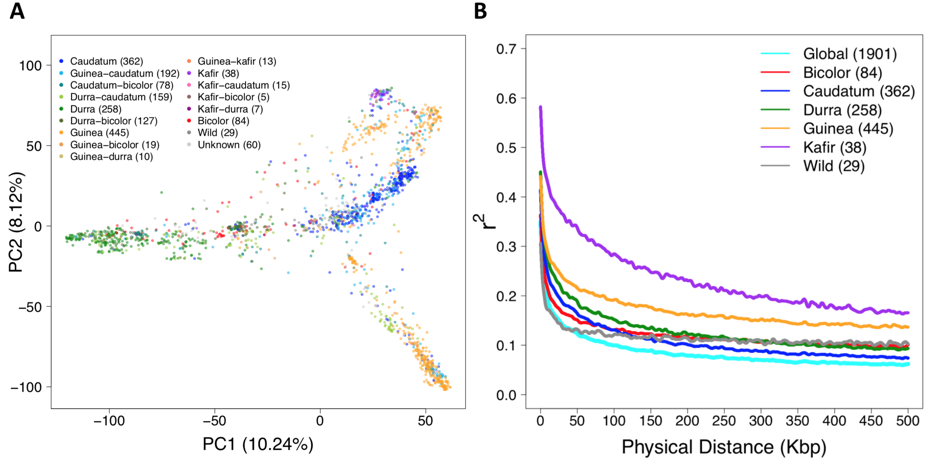


**Figure S2** **Population structure among global sorghum landraces and LD decay patterns among botanical races.** (A) PCA scatter plot of 1901 sorghum landraces. (B) LD decay estimated from 1901 sorghum landraces, five major botanical races and wild relatives. The (A) and (B) panels shared the same color scheme, and landraces were colored according to the sorghum botanical races.

**
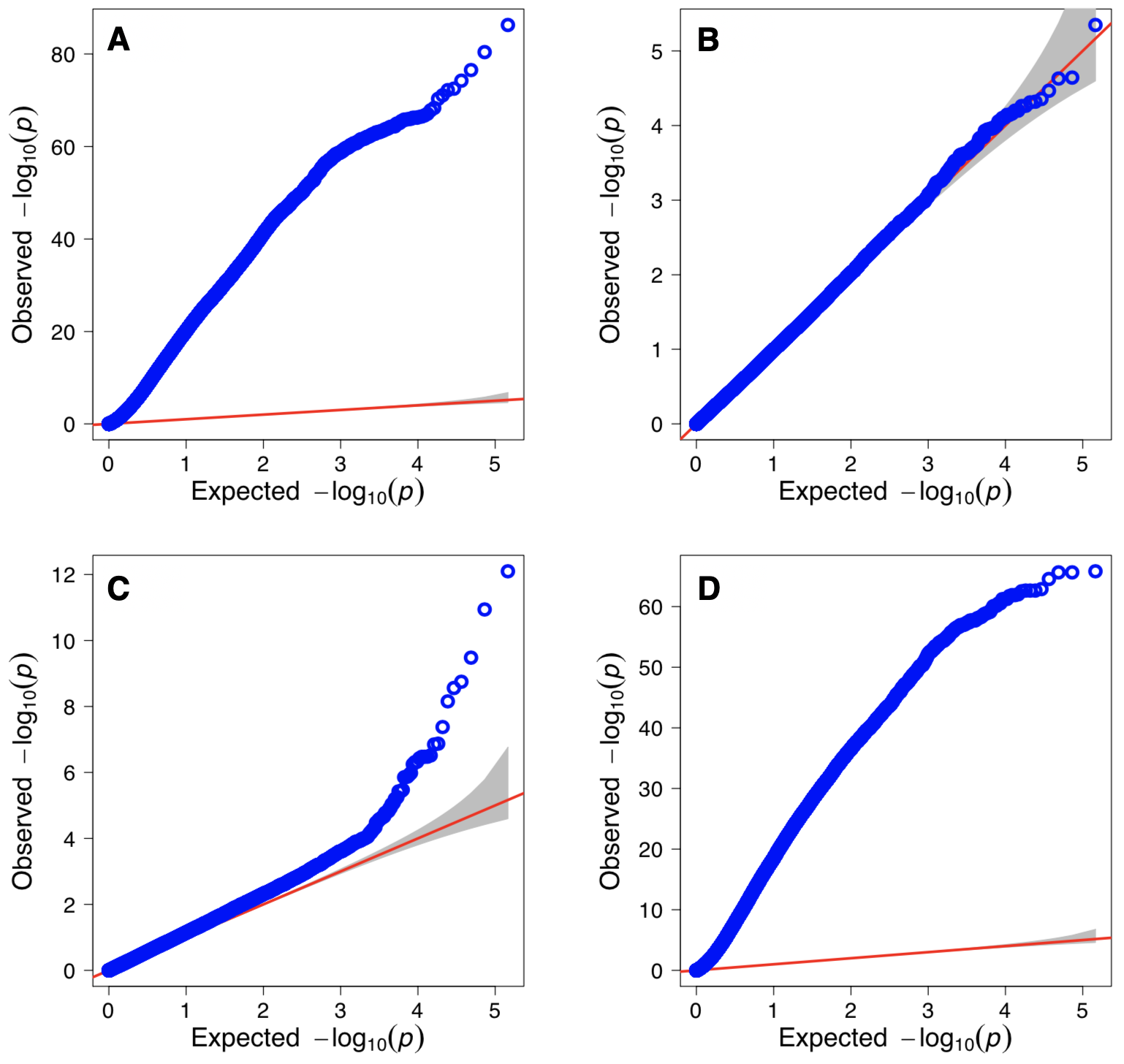
**

**Figure S3 Quantile-quantile (Q-Q) plots of observed versus expected -log_10_(*p*) of the GWAS results.** (A) Naive GLM of 100-seed mass. (B) MLM of 100-seed mass. (C) FarmCPU of 100-seed mass. (D) Naive GLM of Prc.Dry.Q. The red lines represent the distribution of SNPs under the null hypothesis of no SNP associated with the trait. The blue points represent the SNPs that were plotted based on quantile distribution of observed -log_10_ (*p*) on the y-axis versus the quantile distribution of expected -log_10_(*p*) on x-axis. The grey shades represent the 95% confidence interval.

**List of supplemental tables:**

**Supplemental Table S1. 1901 sorghum landraces used in this study.** For each landraces, the accession code, latitude and longitude coordinates, botanical races, precipitation values, 100-seed mass measured from NPGS-GRIN and ICRISAT, and BLUPs of 100-seed mass are listed.

**Supplemental** [**Table S2**](https://drive.google.com/open?id=1jVewuphlkb_lPePkXE_ZOz6Mz1yaVIBV)**. Tukey HSD test results of the 100-seed mass differences between specific pairs of groups.** A total of 41 pairwise botanical race comparisons of 100-seed mass are significant when adjusted *P*-value <0.01.

**Supplemental** [**Table S3**](https://drive.google.com/open?id=1_W5YDbtZDPLamoZ7R_Y6y9sI5kXL9C8Y)**. Summary table of identified 29 *a priori* sorghum 100-seed mass genes through GLM GWAS of 100-seed mass BLUPs.** Genome position of the identified gene on sorghum genome assembly v1.4, the most significantly associated SNP with the gene, SNP genome position, minor allele frequency and *P*-values, the synonym gene name on sorghum genome assembly v3.0, the orthologs, and citation reporting the ortholog are included.

**Supplemental** [**Table S4**](https://drive.google.com/open?id=1DLlvaCFjmTVtFYTK1PomJ-x9ssrlJY5T)**. Summary table of identified 56 *a priori* sorghum 100-seed mass genes through MLM GWAS of 100-seed mass BLUPs.** Genome position of the identified gene on sorghum genome assembly v1.4, the most significantly associated SNP with the gene, SNP genome position, minor allele frequency and *P*-values, the synonym gene name on sorghum genome assembly v3.0, the orthologs, and citation reporting the ortholog are included.

**Supplemental Table S5. Summary table of identified 13 significant SNPs through FarmCPU GWAS of 100-seed mass BLUPs.** SNP genome position on sorghum genome assembly v1.4, minor allele frequency, *P*-values, and SNP effect are included.

**Supplemental Table S6. Summary table of identified 293 sorghum 100-seed mass candidate genes through FarmCPU GWAS of 100-seed mass BLUPs.**  The thirteen significant SNPs identified by FarmCPU, SNP genome position, identified candidate gene, genome position of the identified gene on sorghum genome assembly v1.4, the synonym gene name on sorghum genome assembly v3.0, and the functional annotations of the candidate genes are included.

**Supplemental Table S7. Summary table of identified 36 *a priori* sorghum 100-seed mass genes through association mapping of precipitation in the driest quarter.** Genome position of the identified gene on sorghum genome assembly v1.4, the most significantly associated SNP with the gene, SNP genome position, minor allele frequency and *P*-values, the synonym gene name on sorghum genome assembly v3.0, the orthologs, and citation reporting the ortholog are included.

**Supplemental Table S8. Summary table of identified 35 *a priori* sorghum 100-seed mass genes through SNMF-based *F*_ST_.** Genome position of the identified gene on sorghum genome assembly v1.4, the most significantly associated SNP with the gene, SNP genome position, minor allele frequency and *P*-values, the synonym gene name on sorghum genome assembly v3.0, the orthologs, and citation reporting the ortholog are included.

**Supplemental Table S9. Summary table of identified 37 *a priori* sorghum 100-seed mass genes through PCAdapt.** Genome position of the identified gene on sorghum genome assembly v1.4, the most significantly associated SNP with the gene, SNP genome position, minor allele frequency and *P*-values, the synonym gene name on sorghum genome assembly v3.0, the orthologs, and citation reporting the ortholog are included.

**Supplemental Table S10. 207 colocalized SNPs identified by the GLM GWAS of 100-seed mass and GLM GWAS of Prc.Dry.Q.** Genome position of SNP and *P*-values of GLM GWAS of 100-seed mass and GLM GWAS of Prc.Dry.Q are included.

**Supplemental Table S11. Eleven colocalized SNPs identified by the GLM GWAS of 100-seed mass, GLM GWAS of Prc.Dry.Q and *F*_ST_ and their nearest sorghum genes.** Genome position of SNP and, nearest gene names on sorghum genome assembly v1.4, the synonym gene name on sorghum genome assembly v3.0 and putative functional annotation from MOROKOSHI sorghum transcriptome database.

**Supplemental Table S12. List of QTLs of sorghum seed mass and size reported by previous mapping studies and the QTLs genome positions on sorghum genome assembly v1.4.**
